# The full-length mitochondrial genome of the crocodile icefish, *Chionobathyscus dewitti* (Teleostei: Perciformes: Channichthyidae)

**DOI:** 10.1080/23802359.2019.1624635

**Published:** 2019-07-11

**Authors:** Keun-Yong Kim, Moongeun Yoon, Jong Su Yoo, Dae-Sung Lee

**Affiliations:** a AquaGenTech Co., Ltd, Busan, Republic of Korea;; b National Marine Biodiversity Institute of Korea, Seocheon, Republic of Korea

**Keywords:** *Chionobathyscus dewitti*, crocodile fish, mitochondrial genome, phylogeny

## Abstract

The full-length mitochondrial genome of the crocodile icefish, *Chionobathyscus dewitti* (Teleostei: Perciformes: Channichthyidae) was analyzed by the primer walking method. The mitogenome was 17,451 bp in total length, comprising 13 protein-coding genes, two ribosomal RNA genes, and 22 transfer RNA genes. Its gene order was congruent with those of the other crocodile icefish but different with those of typical vertebrates. In the phylogenetic tree, *C. dewitti* showed the closest relationship to *Chaenocephalus aceratus* in the same family.

Crocodile icefish belonging to the family Channichthyidae (Teleostei: Perciformes) is also known as white-blooded icefish owing to their distinct ecological niche of subfreezing Antarctic Oceans and their unique physiological feature of lacking hemoglobins, the oxygen-binding protein in vertebrate blood (Sidell et al. 1997). Among them, *Chionobathyscus dewitti* occurs in the deep-sea (up to 2000 m) around Antarctic waters (Balushkin and Prut’ko 2006; Sutton et al. 2008). Thus, its biology and taxonomy are poorly studied (Iwami and Kock 1990; Voskoboinikova and Aibulatov 2005). In this study, we analyzed the full-length mitochondrial genome of *C. dewitti* for the first time and reconstructed the phylogenetic tree to reveal its relationship among crocodile icefish.

A specimen of *C. dewitti* was caught by an observer as a by-catch of fisheries targeting the Antarctic krill (*Euphausia superba*) in the South Ocean in 2015 (N 72°25′00″ E 176°04′00″) and deposited in the National Marine Biodiversity Institute of Korea (MABIK Lot No. 0000077). The genomic DNA was extracted from its fin tissue according to Asahida et al. (1996). The mitogenome was amplified through two independent and overlapping PCR runs, and the PCR products were completely sequenced using a set of 25 sequencing primers (oligonucleotide information available upon request). The sequence was deposited in the GenBank under the accession number DO526431.

All mitogenome sequences of the species belonging to the family Channichthyidae were retrieved from GenBank in NCBI (http://www.ncbi.nlm.nih.gov/). They were aligned together with the *C. dewitti* sequence and refined manually to correct obvious misalignments. The nucleotide matrix of 12 protein-coding genes, excluding *nad6* gene, two ribosomal RNA, and 22 transfer RNA genes was created. For the phylogenetic analysis, the matrix was divided into five partitions, consisting of 3620, 3620, 3620, 2631, and 1556 bp for first, second and third bases of each codon of protein-coding genes, and two structural RNA genes, respectively. The alignment information is available upon request in FASTA format. Phylogenetic tree was reconstructed using RAxML 7.0.4 (Stamatakis 2006) for maximum-likelihood (ML) analysis.

The *C. dewitti* mitogenome was a circular molecule of 17,451 bp in total length, consisting of 13 protein-coding genes, two ribosomal RNA genes, and 22 transfer RNA genes, of which gene content is identical to those of typical vertebrates. Its gene order was identical to those of corresponding mitochondrial genomes that Antarctic icefish have (Lee et al. 2015; Song et al. 2016), but different to those of typical vertebrates have, by showing the translocated order between *cob-trnT* and *nad6-trnE* genes.

With the full-length mitogenome sequence of *C. dewitti* analyzed in this study, a phylogenetic tree was reconstructed by the ML method, using the nucleotide sequence matrix from 12 concatenated protein-coding genes and two structural RNA genes (Figure 1). In the resulting tree, all crocodile icefish species were consistently clustered together with respect to the outgroup, *Parachaenichthys charcoti* (Bathydraconidae). Among the lineage, *Champsocephalus gunnari* placed at the most basal position and was separated from the other crocodile icefish. Thereafter, *C. dewitti* showed the closest relationship to *Chaenocephalus aceratus* and separated from *Chaenodraco wilsoni* and three *Chaenodraco* species.

**Figure 1.**
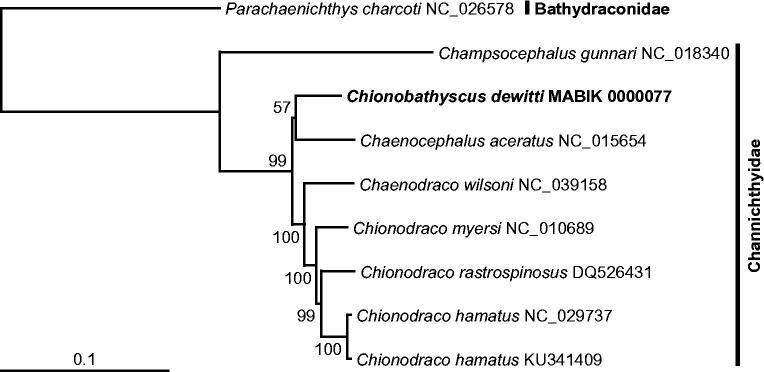
Maximum-likelihood (ML) phylogeny based on the full-length mitochondrial genomes from the crocodile icefish belonging to the family Channichthyidae (Teleostei: Perciformes). The nucleotide sequence matrix included the three codon positions of the 12 protein-coding genes and two structural RNA genes. A bootstrap value above 50% in the ML analysis is indicated at each node. *Chionobathyscus dewitti* analyzed in this study is shown in bold.

In this study, the complete mitochondrial genome of *C. dewitti* was analyzed for the first time, and the phylogenetic tree among crocodile icefish was reconstructed with inference to the genetic data to reveal its phylogenetic relationship. Our study will provide baseline data for its population genetics and conservation strategies for natural living resources including *C. dewitti* in the Antarctic Oceans.
